# Synergistic chemo‐enzymatic hydrolysis of poly(ethylene terephthalate) from textile waste

**DOI:** 10.1111/1751-7915.12734

**Published:** 2017-06-02

**Authors:** Felice Quartinello, Simona Vajnhandl, Julija Volmajer Valh, Thomas J. Farmer, Bojana Vončina, Alexandra Lobnik, Enrique Herrero Acero, Alessandro Pellis, Georg M. Guebitz

**Affiliations:** ^1^ Department of Agrobiotechnology IFA‐Tulln University of Natural Resources and Life Sciences Vienna Inst. of Environ. Biotech. Konrad Lorenz Strasse 20 3430 Tulln a. d. Donau Austria; ^2^ Laboratory for Chemistry and Environmental Protection Institute of Engineering Materials and Design Faculty of Mechanical Engineering University of Maribor Smetanova ulica 17 2000 Maribor Slovenia; ^3^ Green Chemistry Centre of Excellence Department of Chemistry University of York Heslington York YO10 5DD UK; ^4^ Austrian Centre of Industrial Biotechnology Division Polymers & Enzymes Konrad Lorenz Strasse 20 3430 Tulln a. d. Donau Austria

## Abstract

Due to the rising global environment protection awareness, recycling strategies that comply with the circular economy principles are needed. Polyesters are among the most used materials in the textile industry; therefore, achieving a complete poly(ethylene terephthalate) (PET) hydrolysis in an environmentally friendly way is a current challenge. In this work, a chemo‐enzymatic treatment was developed to recover the PET building blocks, namely terephthalic acid (TA) and ethylene glycol. To monitor the monomer and oligomer content in solid samples, a Fourier‐transformed Raman method was successfully developed. A shift of the free carboxylic groups (1632 cm^−1^) of TA into the deprotonated state (1604 and 1398 cm^−1^) was observed and bands at 1728 and 1398 cm^−1^ were used to assess purity of TA after the chemo‐enzymatic PET hydrolysis. The chemical treatment, performed under neutral conditions (T = 250 °C, P = 40 bar), led to conversion of PET into 85% TA and small oligomers. The latter were hydrolysed in a second step using the *Humicola insolens* cutinase (HiC) yielding 97% pure TA, therefore comparable with the commercial synthesis‐grade TA (98%).

## Introduction

Global population and rising living standards are directly correlated to the continuous increase of textile waste (Allwood *et al*., 2006). Overproduction of fabrics since 2010 is driven by the increased rate of replacement of products. In 2008, around 14 M tons of textile waste was generated in Europe, but only 5 M tons was recovered (Zamani, 2014). About 75% of the recovered fabrics were reused or recycled mainly in industrial applications (Jorgensen *et al*., 2008). The remaining collected waste textiles are either landfilled or incinerated. However, the recycling of used textiles would lead to several environmental benefits such as energy saving, as this process requires less energy than the production of the same products from virgin materials and reduction of the carbon footprint of the overall process (Clark *et al*., 2016).

The textile and clothing industry is a heterogeneous business which covers different types of fibres, with a consistent 54% that is represented by man‐made synthetic materials. The consumption of these synthetic fibres increased by 77% between 2000 and 2012 (Harder *et al*., 2017). On the other hand, the growth share of synthetic fibres in global consumption results in the rising demand for petroleum‐based chemicals (Pellis *et al*., 2016a). Among the synthetic textiles, poly(ethylene terephthalate) (PET) is one of the most widely used polymers in the global textile industry (Herrero Acero *et al*., 2011). PET is a semi‐crystalline thermoplastic polymer which shows excellent tensile strength, chemical resistance and high thermal stability. Two PET grades are dominating the global market: the fibre grade PET, with a M_w_ of 15–20 Kg mol^−1^ and intrinsic viscosity between 0.4 and 0.75 dl g^−1^, and the bottle grade PET, which refers to a higher M_w_ polymer (> 20 Kg mol^−1^) with an intrinsic viscosity above 0.95 dl g^−1^ (Tasca *et al*., 2010; Al‐Sabagh *et al*., 2016).

Due to its wide production and utilization (Cavaco‐Paulo and Guebitz, 2008), PET represents a broad disposal inert textile. The non‐toxic nature, durability and crystal clear transparency of PET during use are the principal advantages of this polyester, while its rather slow biodegradability is the major cause of concern to the environmentalists. Recycling the textile waste‐derived polyesters can significantly cut down the energy usage, resource depletion and greenhouse gas emissions. Unfortunately, different factors such as colouring dyes (Giannotta *et al*., 1994) and other chemicals such as detergents, fuels and pesticides (Demertzis *et al*., 1997) reduce the quality of recycled PET reducing the number of the possible applications. When compared to mechanical recycling and incineration, chemical hydrolysis could lead to higher value products (Paszun and Spychaj, 1997). The most common chemical‐based PET hydrolysis processes are alkaline hydrolysis using 4–20% NaOH/KOH solutions (Karayannidis *et al*., 2002), phase transfer catalysts and acidic hydrolysis using concentrated sulphuric acid or other mineral acids (Yoshioka *et al*., 2001). All these processes are very costly and toxic because the chemicals required and laborious purification steps are needed. In recent years, more environmentally friendly PET recycling strategies based on neutral hydrolysis (carried out using water or steam at 1–4 MPa and temperatures of 200–300 °C) were reported (Launay *et al*., 1994). In last decade, the interest of biotechnologies towards polyesters biodegradation and recycling is gaining a key role. Yoshida *et al*. (2016) showed a novel bacterium, *Ideonella sakaiensis* 201‐F6, able to break down the plastic using two enzymes to hydrolyse PET and assimilate its building block for growth. Earlier, various studies demonstrated that a class of enzymes belonging to the α/β hydrolase family, namely cutinases, are able to hydrolyse the ester bonds of PET and several other polyesters (Pellis *et al*., 2015, 2016b; Barth *et al*., 2016; Wei *et al*., 2016). Among them, cutinase are currently under investigation for the bioprocessing of PET textiles on an industrial scale (Silva *et al*., 2005). Earlier, it was reported that cutinases from *Thermobifida fusca* and *Humicola insolens* were able to hydrolyse low‐crystallinity PET while complete hydrolysis by enzymes only seems to be difficult if not impossible for PET with higher crystallinity (Mueller, 2006; Nimchua *et al*., 2007; Ronkvist *et al*., 2009). In this work, we propose an innovative synergistic chemo‐enzymatic hydrolysis of PET for the production of high‐purity TA (97%) avoiding harsh chemical treatments.

## Results and discussion

### FT‐Raman analysis

In the past, several methods, mainly HPLC‐related, have been established to follow enzymatic hydrolysis of PET in aqueous solutions (Herrero Acero *et al*., 2011; Pellis *et al*., 2016c). However, water‐insoluble oligomers would obviously escape quantification of TA in the presence of insoluble oligomers; hence, a novel method based on FT‐Raman and triethylamine was adapted for this study. TA, *p*‐C_6_H_4_(COOH)_2_, has two carboxylic groups which are detectable using Fourier transform Raman spectroscopy. The analysis of benzene dicarboxylic acids such as isophthalate and terephthalate using FT‐Raman was previously described by Arenas and Tellez (Arenas, 1979; Téllez *et al*., 2001). According to these reports, the ‐COOH group of solid TA reveals a typic‐centred band at 1631 cm^−1^, mainly given by the asymmetric stretching (ν_as_) of C=O. As described by Tellez, a coupled vibrational mode is characteristic of the single band at 1286 cm^−1^. The discrimination of monomeric TA from esterified species was performed converting these functional groups into the corresponding anions. The simplest reaction of carboxylic acid is salification by a base. This reaction causes the shift of (ν_as_) of the C=O group and appearance of new bands due to the in‐phase and out of phase ‐COO^(−)^ stretching vibration (Socrates, 2004). The shift of the acid peak of the carboxylic group from 1720 cm^−1^ to 1580 cm^−1^ after alkaline treatment was previously reported for the grafting of cotton with cyclodextrins (Voncina and Majcen, 2005) or for detection of end groups of fluoropolymers (Pianca *et al*., 1999).

The conversion of the ‐COOH group into the carboxylate species was carried out using triethylamine (TEA) in chloroform solution (Di Robert *et al*., 2015). The deprotonated anion obtained *via* incubation with tertiary aliphatic amine caused the shift of ν_as_C=O and the increase of the single band of the symmetric stretching (ν_s_) of C=O at 1400 cm^−1^ and an ammonium‐related band in the 2700 cm^−1^ region.

**Figure 1:**
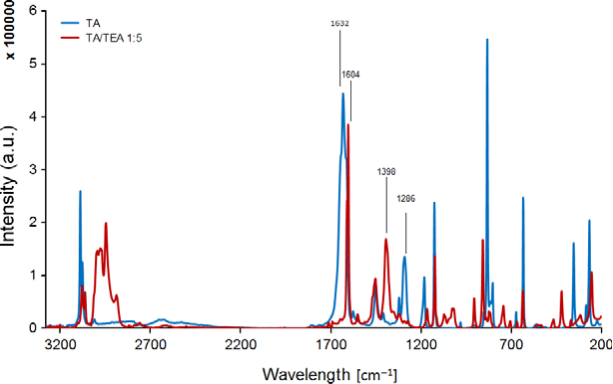
FT‐Raman analysis showing the deprotonation of the TA's carboxylic acid moieties (blue) *via* incubation with a 1:5 TA/TEA ratio (red). Spectra were normalized between the region 2000 and 2200 cm^−1^.

Figure 1 shows the shift of the acid peak of solid TA from 1631 to 1604 cm^−1^ after deprotonation with TEA. Furthermore, the clear appearance of a peak at 1398 cm^−1^ was observed due the symmetric stretching mode of the carboxylate moiety. On the other hand, the peak at 1286 cm^−1^ was strongly reduced. The 1:5 TA/TEA ratio showed the clearest signal for the monomers due to a complete conversion of the desired groups (Fig. S2).

To assess the suitability of this method to quantify TA in the presence of oligomers, BHET, DMT and PET were incubated with the tertiary aliphatic amine (1:5 ratio). Expectedly, the shift of 1632 and 1395 cm^−1^ typical for TA did not occur as all carboxylic groups of these oligomers are esterified (Figs S3–S5).

### Water‐based PET hydrolysis

Preliminary experiments showed that water‐based PET depolymerization was not achieved with ratio 1:4 PET/H_2_O and incubation for 90 min. Therefore, a series of experiments were performed, to define the appropriate depolymerization conditions (Table 1).

**Table 1:** Design of experiments strategy used for performing the water‐based hydrolysis of PET

Experiment	Initial MW (IV) range of PET	T [°C]	PET/H_2_O ratio^a^	Steady‐state time [min]^b^
1	0.62	180	1/4	0
2			1/10	30
3		250	1/4	
4			1/10	0
5		180	1/4	
6			1/10	30
7		250	1/4	
8			1/10	0

All reported conditions were also tested; zinc acetate was added to the reaction mixture.

**a.** 25 g of PET.

**b.** After the transient period (to reach the desired T).

The incubation of PET fibre at 180 °C and 12 bar did not lead to any depolymerization of the sample, and hence, there was no difference of spectra. In Fig. S6, it is possible to observe how the spectra of raw PET fibre and Sample 2 are very similar with no remarkable differences that could be spotted, showing that reaction temperature and pressure were not optimal for the hydrolysis of the polymer. Finally, when temperature and pressure were increased to 250 °C and 39 bar, respectively, the polymer was completely reduced in a whitish powder (Fig. S7). In Fig. S8, it is possible to see that spectrum of PET depolymerization product for Sample 4 (blue) was similar to the spectrum of commercial TA. Using these conditions, a powder consisting of 85% TA was obtained from the water‐based PET depolymerization according to HPLC and ^1^H‐NMR analysis. Unfortunately, further variation of the reaction conditions including addition of zinc acetate did not lead to a higher yield of TA. Hence, enzymatic hydrolysis of the remaining oligomers was assessed in the next step. To note that experiments with various steady‐state lengths were performed but did not lead to an improvement of the TA yield, therefore the enzymatic finishing step was critical in order to obtain high‐purity TA.

### Enzymatic PET hydrolysis

The kinetics of the formation of degradation products *via* enzymatic treatment depends on various factors, including the chemical–physical structure of polyester substrate (Eberl *et al*., 2008). Already, various authors have previously demonstrated that cutinase preferentially hydrolyze the amorphous region on PET (Donelli *et al*., 2009; Gamerith *et al*., 2017). On the other hand, it was shown that enzymatic hydrolysis of PET oligomers is way faster than of a long‐chain polymer (Ribitsch *et al*., 2011). Therefore, due to the high crystallinity of the PET fibres used in the textile industry, a chemical pre‐hydrolysis was established in this work.

**Figure 2:**
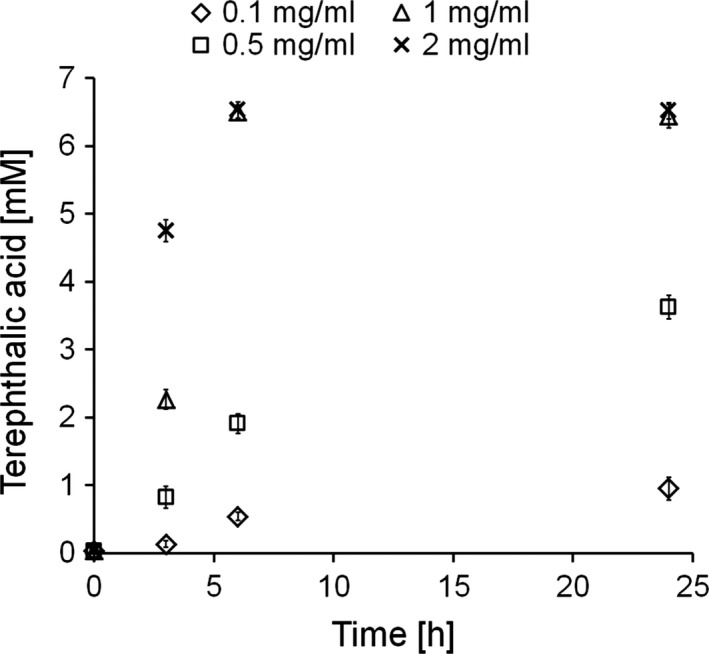
Enzymatic hydrolysis of chemically pre‐treated PET with different concentrations of *Humicola insolens* cutinase (HiC).

The sample from chemical pre‐treatment with the highest degree of hydrolysis (Sample 4, 85% TA) was further incubated with different concentrations of *Humicola insolens* cutinase (HiC) to hydrolyse remaining oligomers (Fig. 2). The highest amount of soluble TA (6.5 mM) was obtained after 6 h of incubation both when 1 or 2 mg ml^−1^ of HiC was applied without any further increase until 24 h of incubation. The lower concentrations of enzyme of 0.1 and 0.5 mg ml^−1^ led to the release of 0.53 and 1.9 mM of TA respectively.

**Figure 3:**
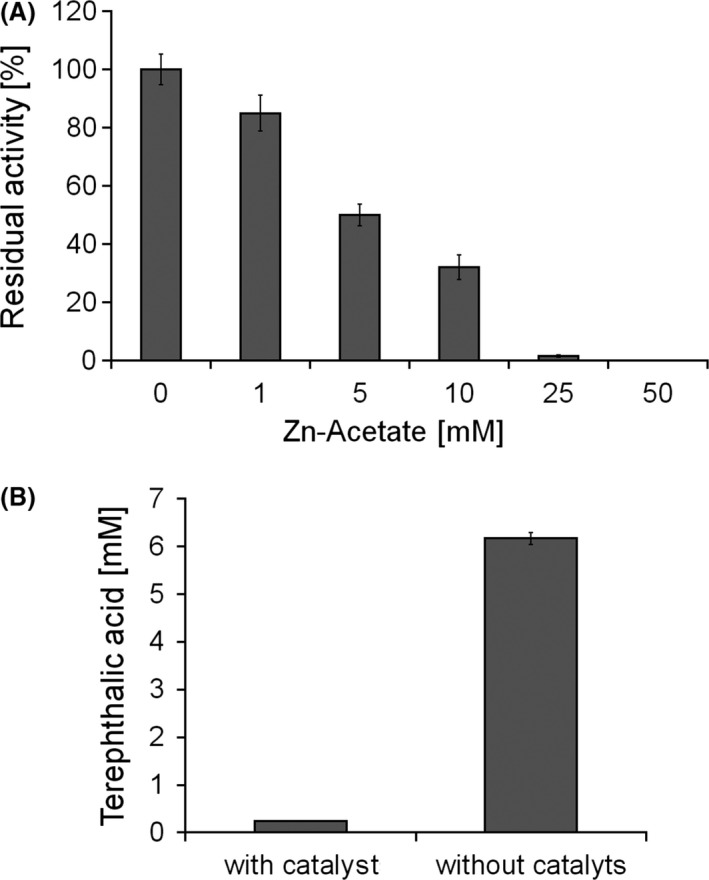
Residual activity of HiC in the presence of Zn acetate (A) and enzymatic hydrolysis of PET chemically pre‐treated in the presence of Zn acetate (B).

Terephthalic acid with 97% purity was obtained after enzymatic treatment of the chemical‐treated PET which is comparable to synthesis‐grade TA (98% pure). When chemical pre‐hydrolysis of PET was performed in the presence of zinc acetate as a catalyst, a negative influence on enzymatic hydrolysis was observed (Fig. 3). In fact, there was no further increase in the amount of TA seen (Fig. 3).

Zinc and other metal ions are well known to be potential inhibitors of many enzymes, including cutinases as recently reported by Chen *et al*. that biochemically characterized *Thermobifida fusca* cutinases (Chen *et al*., 2010).

### FT‐Raman detection of depolymerized PET

**Figure 4:**
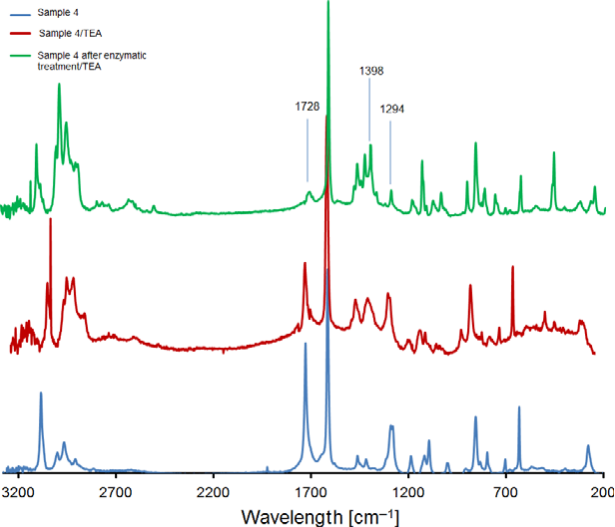
FT‐Raman analysis showing the incubation of Sample 4 with a 1:5 TEA ratio before (red) and after (green) enzymatic hydrolysis. Spectra were normalized between the 2000 and 2200 cm^−1^ region.

**Figure 5:**
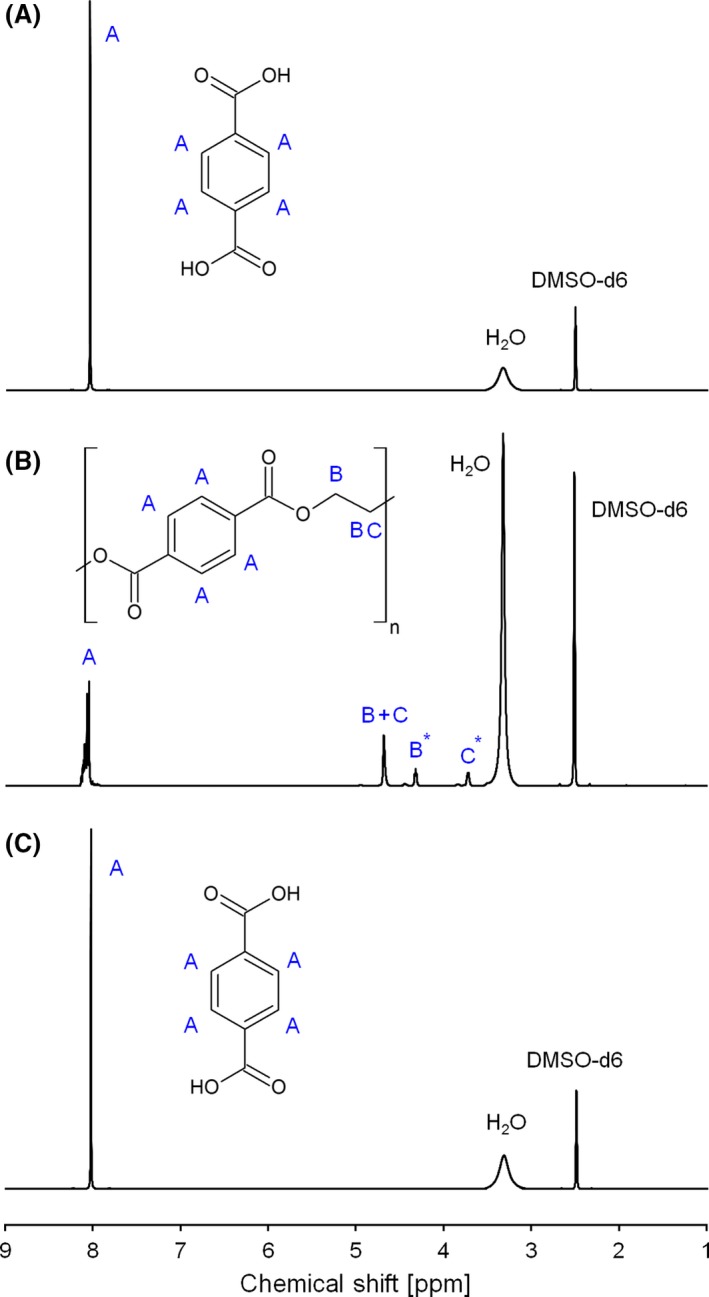
^1^H‐NMR of pure TA (A), PET degradation after the chemical treatment (Sample 4, B) and PET degradation after enzymatic finishing (C). All spectra were recorded in DMSO‐d_6_. All samples were fully soluble in the selected solvent. For detailed proton assignments, please see ESI.

While in the chemically pre‐treated sample oligomers were present, the peak at 1728 cm^−1^ indicative of ester bond was considerably reduced after enzymatic hydrolysis (Fig. 4). In parallel, the increase in the band at 1398 cm^−1^ indicated formation of TA. Finally, the signal at 1286 cm^−1^, as expected, had the opposite trend of the asymmetric stretching of CO, further confirming the reduction in the oligomers to TA (Fig. 5).

In addition to FT‐Raman, ^1^H‐NMR analysis was performed. Likewise, ^1^H spectra (recorded in DMSO‐d_6_) indicate the presence of some longer oligomers in the chemically pre‐treated sample while the spectra of the reaction product after subsequent enzymatic hydrolysis suggest their conversion into TA. Based on the ^1^H‐NMR‐performed calculations (see ESI for details), the oligomers are mostly comprised of less than four (but nearer two) constitutional repeat units, and TA end groups dominate over EG suggesting the oligomerization is more effective at releasing free EG than free TA, or that the purification steps involved preferentially remove EG.

## Materials and Methods

### Chemicals, substrates and enzymes

Buffer components, bovine serum albumin (BSA), *para*‐nitrophenyl‐butyrate (*p*‐NPB), methanol, zinc acetate, TA and formic acid were purchased from Sigma‐Aldrich (USA). All other chemicals and reagents used in this work were of analytical grade and used without further purification if not otherwise specified. *Humicola insolens* cutinase (HiC) was a gift from Novozymes (Beijing, China). All hydrolyses were performed using Wellman PET fibre with a viscosity of 0.62 dl g^−1^.

### Water‐based PET hydrolysis

Water‐based PET hydrolysis was performed in 1 l stainless steel high‐pressure and high‐temperature reactor at different temperatures (180 and 250 °C) and with and without the addition of zinc acetate as catalyst. All experiments were carried out with 25 g of virgin PET fibre in 250 ml of deionized water. The reactions were stopped after 60 and 90 min, respectively (30 min after reaching the steady state), and two different ratios PET/H_2_O were assessed. A temperature and pressure profile of a typical reaction is shown in Fig. S1. At the end of the process, H_2_O and EG were removed and a white powder was obtained (Fig. S7B). The EG stream can be further processed anaerobically due to the current low cost of the compound.

### ATR FT‐IR analysis

ATR FT‐IR spectra were recorded on a Perkin‐Elmer Spectrum GX spectrometer. The ATR accessory (supplied by Specac Ltd., UK) contained a diamond crystal. A total of 16 scans for each sample were taken with a resolution of 4 cm^−1^. All spectra were recorded at 21 °C over the wavelength interval between 4000 and 650 cm^−1^.

### FT‐Raman analysis

To remove the water, terephthalic acid (TA), bis(2‐hydroxyethyl) terephthalate (BHET), dimethyl terephthalate (DMT) and poly(ethylene terephthalate) PET as standards were lyophilized for 24 h; 50 mg of samples was incubated with different volumes of pure triethylamine in 10 ml of chloroform for 6 h. Afterwards, chloroform was removed by evaporation at 21 °C for 24 h. The virgin material and the powders obtained from the chemical process and after the enzymatic treatment were also incubated as described above. The FT‐Raman spectra were recorded using a Perkin‐Elmer Raman station 400, coupled with a 785 nm laser. Spectra were collected at a resolution of 2 cm^−1^ for 25 scans and normalized in the region 2200–2400 cm^−1^ before any data processing. The bands were assigned as follows: 3000–2700 cm^−1^ ν(Et‐NH_4_^+^), 1728 cm^−1^ ν(C=O, bended), 1632–1604 cm^−1^ ν_as_(C=O), single band 1398 cm^−1^ δ(C‐O), single band 1286 cm^−1^ coupled mode ν(C=O)+δ(COH).

### Protein quantification and SDS‐PAGE analysis

Protein concentration was measured using the Bio‐Rad Protein Assay Kit (Bio‐Rad, Vienna, Austria). BSA was used as protein standard; 10 μl of the sample was added into the wells of a 96‐well plate. Afterwards, 200 μl of the prepared Bio‐Rad reagent solution was added (Bio‐Rad Reagent diluted 1:5 with MQ‐water). The plate was incubated for 5 min at 21 °C and 400 rpm. Buffer (100 mM Tris‐HCl pH 7) was used as blank. The absorption after 5 min was measured at λ = 595 nm in a plate reader (Tecan INFINITE M200) and the concentration calculated from the average of triplicates. Sodium dodecyl sulphate–polyacrylamide gel electrophoresis (SDS‐PAGE) was performed according to Laemmli using 4% stacking gels and 15% separating gels and run at 150 V (Laemmli, 1970). Pre‐stained protein marker IV (Peqlab, Germany) was used as a molecular mass marker. Proteins were stained with Coomassie method.

### Esterase activity assay

Esterase activity was measured at 25 °C using *p*‐nitrophenyl‐butyrate (*p*‐NPB) as a substrate according to Biundo *et al*. (Biundo *et al*., 2015). The final assay was carried out by mixing 200 μl of the substrate stock solution, in 100 mM Tris–HCl buffer pH 7, with 20 μl of enzyme solution. The increase in the absorbance at 405 nm due to the release of *p*‐nitrophenol (ε 405 nm = 10.27 ml (μmol cm)^−1^) was measured for 5 min, every 18 s with a plate reader. A blank was included using 20 μl of buffer instead of enzyme solution. The activity was calculated in units (U), where 1 unit is defined as the amount of enzyme required to hydrolyse 1 μmol of substrate per minute under the given assay conditions. The activity assay was also performed in the presence of 1, 5, 10, 25 or 50 mM of the chemical catalyst (zinc acetate). All the experiments were performed in triplicates.

### Enzymatic hydrolysis of PET oligomers

The enzymatic hydrolysis of the PET powder resulting from the chemical treatments was performed as previously described with some modifications (Herrero Acero *et al*., 2011; Pellis *et al*., 2016a). Briefly, 10 mg of pre‐treated PET powder and 2.0 ml of enzyme solution (diluted in 100 mM Tris‐HCl buffer pH 7) were added in a test tube. The mixture was incubated for 24 h at 50 °C and 600 rpm in an orbital shaker. Different concentrations 0.1, 0.5, 1 and 2 mg ml^−1^ of enzyme were used to understand the optimal concentration of HiC. All the experiments were performed in triplicates.

### High‐Performance Liquid Chromatography

Proteins were removed using a 1:1 (v v^−1^) ice‐cold methanol precipitation. Therefore, samples were centrifuged at 14 000 rpm (Centrifuge Beckman JU‐MI) at 0 °C for 15 min. The supernatant was acidified by adding 8 μl of 6 M HCl and then transferred into HPLC vials. The released products were analysed by high‐performance liquid chromatography (HPLC) (Agilent Technologies, Palo Alto, CA) coupled with a UV detector, at 241 nm, using a water/methanol linear gradient (Table S1). To determine the TA percentage in the mixture, 10 mg of PET powder resulting from before and after the enzymatic hydrolysis was diluted in 100 ml of 100 mM Tris‐HCl buffer pH 7 and analysed as described before.

### ^1^H‐NMR analysis

^1^H nuclear magnetic resonance was performed on a Bruker Avance II 400 spectrometer (resonance frequency of 400.13 MHz for ^1^H) equipped with a 5 mm observe broadband probe head (BBFO) with z‐gradients. DMSO‐*d*_*6*_ was used as NMR solvent for all samples.

## Conclusions

In this study, the synergism of chemical and enzymatic hydrolysis of PET was demonstrated on a multigram scale. To monitor the PET hydrolysis and formation of TA, a FT‐Raman method based on the deprotonation of the –COOH group was established. The chemical pre‐treatment performed in an environmentally friendly way under neutral conditions, therefore avoiding harsh chemicals, leads to depolymerization of the polyester‐composed waste textiles yielding about 85% TA. The enzymatic hydrolysis performed in a second reaction step leads to further hydrolysis of the remaining oligomers yielding TA with a purity of 97%. Future studies should consider the chemo‐enzymatic treatment of different PET containing textiles wastes as well as studies on synthesis of PET based on the recovered TA.

## Author contributions

The manuscript was written through contributions of all authors. All authors have given approval to the final version of the manuscript.

## Conflict of interest

None declared.

## Supporting information


**Fig. S1**. Temperature (blue, Y‐axis left) and pressure (red, Y‐axis right) increase according to the reaction time (X‐axis).
**Fig. S2.** FT‐Raman analysis showing the deprotonation of the TA's carboxylic acid moieties *via* incubation with different TA/TEA ratio.
**Fig. S3.** FT‐Raman analysis showing the deprotonation of the BHET *via* incubation with TEA 1:5.
**Fig. S4.** FT‐Raman analysis showing the deprotonation of the DMT *via* incubation with TEA 1:5.
**Fig. S5.** FT‐Raman analysis showing the deprotonation of the PET *via* incubation with TEA 1:5.
**Fig. S6.** FT‐IR spectrum of Sample 1 (black) and spectrum of untreated virgin PET.
**Fig. S7.** Samples obtained by three different depolymerisation operational conditions.
**Fig. S8**. FT‐IR spectra of Sample 4 (blue), untreated virgin PET (black) and pure TA (red).
**Fig. S9.** SDS‐PAGE of HiC. Lane 1 Protein Marker IV (bands 10‐170 KDa). Lane 2 HiC (dilution 1:10), MW ~24 KDa.
**Table S1.** Mobile phase gradient used for the HPLC‐DAD analysis of the PET degradation release products.
