# Supplementary material for: Synergistic chemo‐enzymatic hydrolysis of poly(ethylene terephthalate) from textile waste
Source: Microb Biotechnol. 2017 Jun 2;10(6):1376–83. doi: 10.1111/1751-7915.12734 (PMC5658601; doi:10.1111/1751-7915.12734)
Supplement: Supplementary file 1 — Fig. S1. Temperature (blue, Y‐axis left) and pressure (red, Y‐axis right) increase according to the reaction time (X‐axis). Fig. S2. FT‐Raman analysis showing the deprotonation of the TA's carboxylic acid moieties via incubation with different TA/TEA ratio. Fig. S3. FT‐Raman analysis showing the deprotonation of the BHET via incubation with TEA 1:5. Fig. S4. FT‐Raman analysis showing the deprotonation of the DMT via incubation with TEA 1:5. Fig. S5. FT‐Raman analysis showing the deprotonation of the PET via incubation with TEA 1:5. Fig. S6. FT‐IR spectrum of Sample 1 (black) and spectrum of untreated virgin PET. Fig. S7. Samples obtained by three different depolymerisation operational conditions. Fig. S8. FT‐IR spectra of Sample 4 (blue), untreated virgin PET (black) and pure TA (red). Fig. S9. SDS‐PAGE of HiC. Lane 1 Protein Marker IV (bands 10‐170 KDa). Lane 2 HiC (dilution 1:10), MW ~24 KDa. Table S1. Mobile phase gradient used for the HPLC‐DAD analysis of the PET degradation release products. [file MBT2-10-1376-s001.docx]

Synergic chemo-enzymatic hydrolysis of poly(ethylene terephthalate) from textile waste.

F. Quartinello^(1)^, S. Vajnhandl^(2)^, J. Volmajer Valh^(2)^, T. J. Farmer ^(3)^ B. Vončina^(2)^, A. Lobnik^(2)^, E. Herrero Acero^(3)^, A. Pellis^(1,*)^, G. M. Guebitz^(1,4)^

*^(1)^ University of Natural Resources and Life Sciences Vienna, Dep. of Agrobiotechnology IFA-Tulln, Inst. of Environ. Biotech., Konrad Lorenz Strasse 20, 3430, Tulln a. d. Donau, Austria*

*^(2)^ Laboratory for Chemistry and Environmental protection, Institute of Engineering Materials and Design*, Faculty of Mechanical Engineering, *University of Maribor, Smetanova ulica 17, 2000 Maribor, Slovenia*

*^(3)^ University of York, Department of Chemistry, Green Chemistry Centre of Excellence, Heslington, York, YO10 5DD, UK*

*^(4)^ Austrian Centre of Industrial Biotechnology, Division Polymers & Enzymes, Konrad Lorenz Strasse 20, 3430, Tulln a. d. Donau, Austria*

*^*^ Corresponding author: Alessandro Pellis PhD, tel:* [*+43 1 47654-97447*](tel:+43%201%2047654-97447)*, email:* [alessandro.pellis@boku.ac.at](mailto:alessandro.pellis@boku.ac.at)

**Keywords:** Poly(ethylene terephthalate), chemical hydrolysis, *Humicola insolens* cutinase, sustainable recycling.


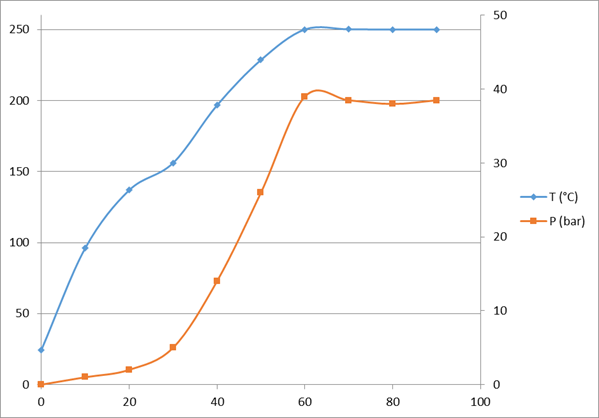


**Figure S1**. Temperature (blue, Y-axis left) and pressure (red, Y-axis right) increase according to the reaction time (X-axis).

| **Table S1**. Mobile phase gradient used for the HPLC-DAD analysis of the PET degradation release products. | | | | | |
| --- | --- | --- | --- | --- | --- |
| Time (min) | H_2_O | Methanol | Formic acid | Flow (mL/min) | Pressure (bar) |
| 1 | 80 | 10 | 10 | 0.75 | 600 |
| 8 | 40 | 50 | 10 | 0.75 | 600 |
| 10 | 0 | 90 | 10 | 0.75 | 600 |


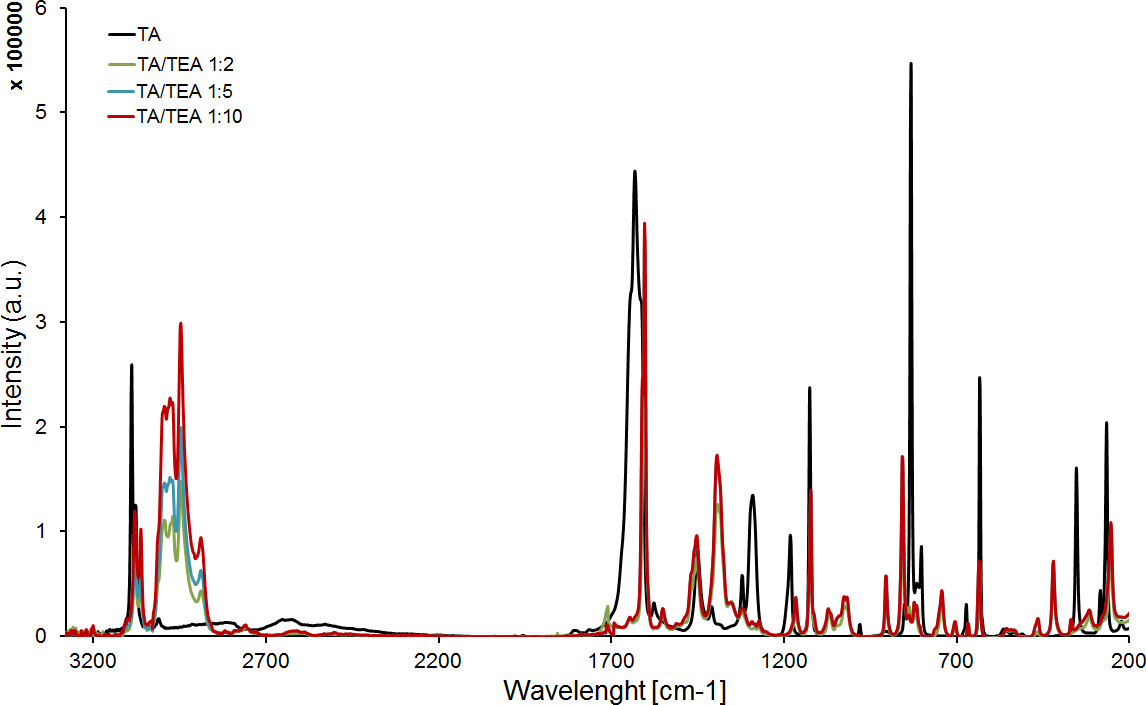


Figure S2. FT-Raman analysis showing the deprotonation of the TA’s carboxylic acid moieties *via* incubation with different TA/TEA ratio


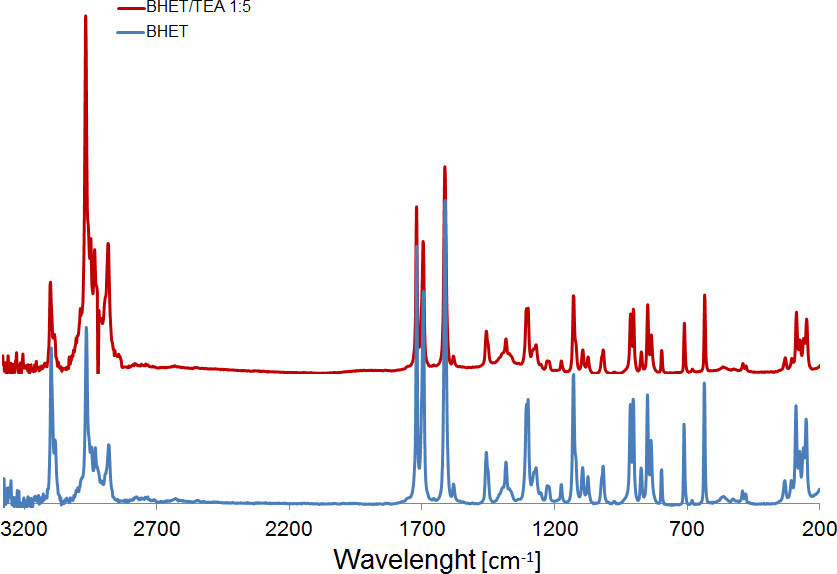


Figure S3. FT-Raman analysis showing the deprotonation of the BHET *via* incubation with TEA 1:5


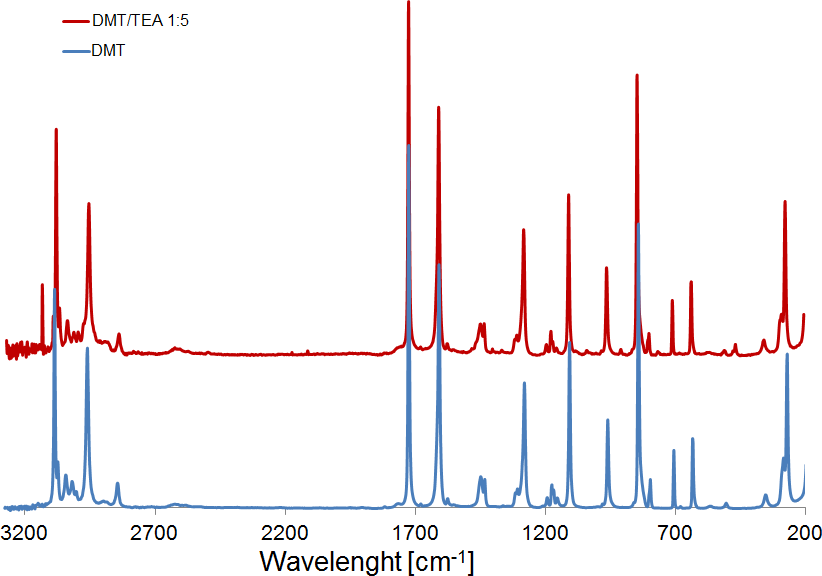


Figure S4. FT-Raman analysis showing the deprotonation of the DMT *via* incubation with TEA 1:5


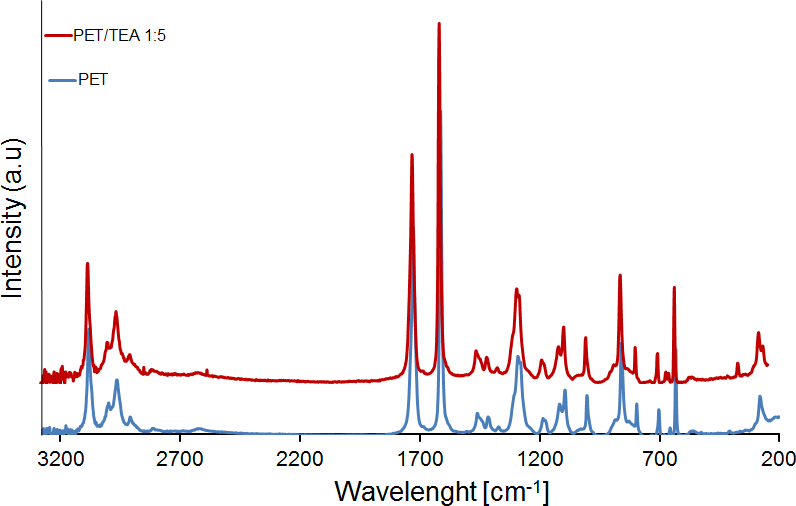


Figure S5. FT-Raman analysis showing the deprotonation of the PET *via* incubation with TEA 1:5


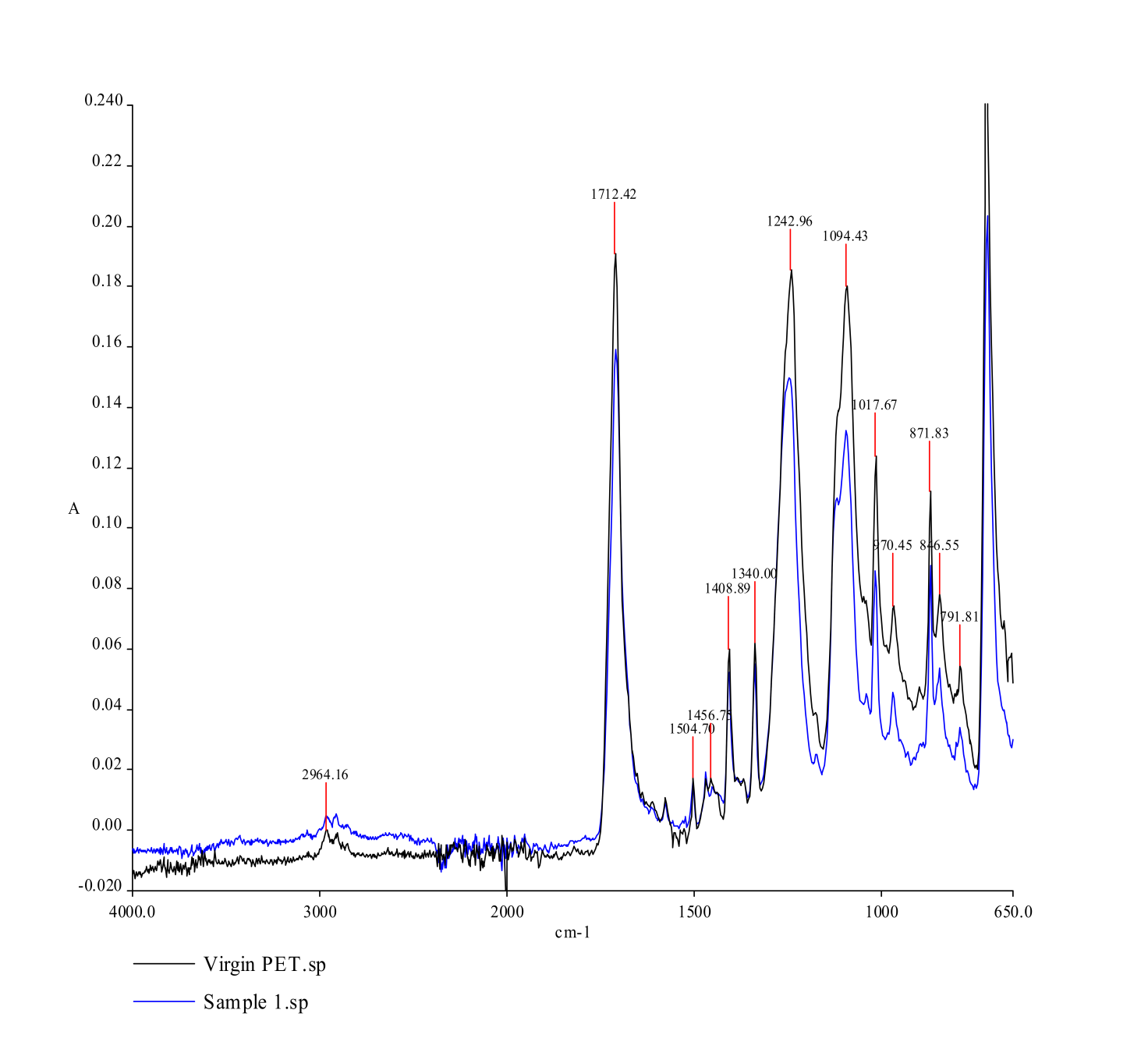


Figure S6. FT-IR spectrum of Sample 1 (black) and spectrum of untreated virgin PET (blue


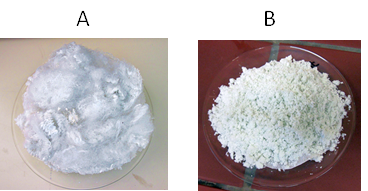


Figure S7. Samples obtained by 3 different depolymerisation operational conditions

A - Sample obtained by experiment: T=180°C, P=12 bars, t=0 min after reaching steady state conditions.

B - Sample obtained by experiment: T=250°C, P=39 bars, t=0 min after reaching steady state conditions


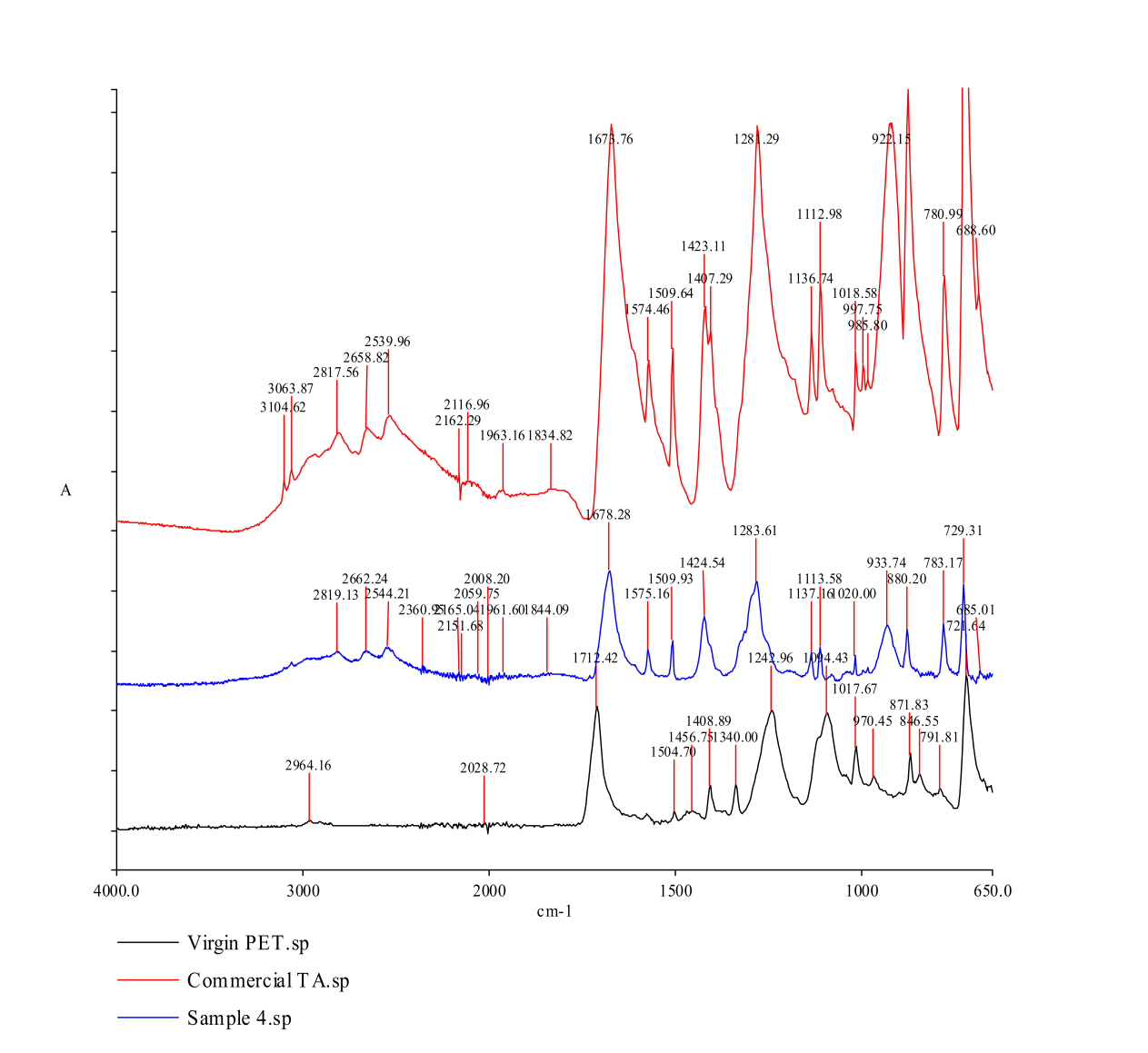


**Figure S8**. FT-IR spectra of Sample 4 (blue), untreated virgin PET (black) and pure TA (red).


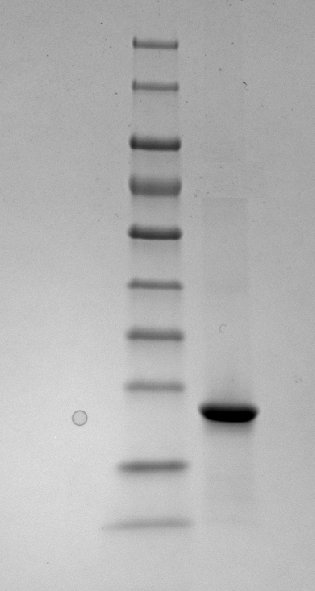


**Figure S9**. SDS-PAGE of HiC. Lane 1 Protein Marker IV (bands 10-170 KDa). Lane 2 HiC (dilution 1:10), M_W_ ̴24 KDa.

**^1^H-NMR calculations on the released products**

| Chemical shift (ppm) | Assignment | Integral | No. of protons | Notes |
| --- | --- | --- | --- | --- |
| 8.05 | A = Ar-H (bulk+end-group) | 4.0 | 4 | Set as reference |
| 4.67 | B+C = EG bulk (ArOC**H2**C**H2**OAr) | 1.69 | 4 |  |
| 4.31 | B* = EG end-group (ArOC**H2**CH2OH) | 0.59 | 2 |  |
| 3.72 | C* = EG end-group (ArOCH2C**H2**OH) | 0.59 | 2 |  |

Note: B = B* and C = C* when EG is an end-group

Ratio of TA to EG:

*[A/4] : [(B+C+B*+C*)/4] = [4.0/4] : [(1.69+0.59+0.59)/4] = 1 : 0.72 TA:EG*

Therefore there is an excess of TA, this being either as oligomers or free TA

End-group Analysis: We are unable to distinguish between the bulk (i.e. within the chain), end-group or free TA units via ^1^H-NMR spectroscopy but based on the above ratio there is a 1 : 0.72 excess of TA relative to EG. This excess must be either as end-groups or free TA therefore ~28% (i.e. 100% - 72%) of the TA units are assumed to be end-groups but virtue of there excess relative to EG.

We can distinguish whether the EG units are bulk or end-group, and would also see free EG but this is not evident in the sample. Therefore a ratio of bulk : end-group EG can be determined:

*EG_bulk_ : EG_end-group_ = [B+C/4] : [(B*+C*)/4] = [1.69/4] : [(0.59+0.59)/4] = 0.42 : 0.30 = 1 : 0.70*

Based on this ratio of 1 : 0.70 EG_bulk_ : EG_end-group_ we can determine that 41% of the EG units are end-groups.

As some of the EG are end-groups despite the excess of TA calculated above there must therefore be additional units of TA that are also end-groups (i.e. those as a result of EG becoming end-groups thus freeing more carboxylic acid end-groups). This can be estimated based on the amounts of EG_end-group_. We had already determined that ~28% of all TA was end-groups due to it being in excess, but a further 41% of the remaining (72%) TA is also end-groups. As 41% of the remaining 72% = 30% then a total of 58% (i.e. 30% + 28%) of all TA must be end-groups or free TA.
